# Understanding the factors affecting the attraction and retention of health professionals in rural and remote areas: a mixed-method study in Niger

**DOI:** 10.1186/s12960-017-0227-y

**Published:** 2017-09-04

**Authors:** Loubna Belaid, Christian Dagenais, Mahaman Moha, Valéry Ridde

**Affiliations:** 1 École de santé publique, département de médecine sociale et préventive de l’Université de Montréal, 7101 Av du Parc, 3e étage, Montréal, Québec H3N 1X9 Canada; 2 Université de Montréal, département de psychologie, Pavillon Marie Victorin, 90 Avenue Vincent D’indy & Boulevard Mont-Royal, Montréal, Québec H2V 2S9 Canada; 3 Laboratoire d’études et recherches sur les dynamiques sociales et le développement local (LASDEL), BP:12901 Niamey, Niger; 4 Institut de recherche en santé publique de l’Université de Montreal (IRSPUM), Montreal, Quebec Canada

## Abstract

**Background:**

The critical shortage of human resources in health is a critical public health problem affecting most low- and middle-income countries, particularly in sub-Saharan Africa. In addition to the shortage of health professionals, attracting and retaining them in rural areas is a challenge. The objective of the study was to understand the factors that influence the attraction and retention of health professionals working in rural areas in Niger.

**Methods:**

A mixed-method study was conducted in Tillabery region, Niger. A conceptual framework was used that included five dimensions. Three data collection methods were employed: in-depth interviews, documentary analysis, and concept mapping. In-depth interviews were conducted with three main actor groups: policy-makers and Ministry of Health officials (*n* = 15), health professionals (*n* = 102), and local health managers (*n* = 46). Concept mapping was conducted with midwifery students (*n* = 29). Multidimensional scaling and cluster analysis were performed to analyse the data from the concept mapping method. A content analysis was conducted for the qualitative data.

**Results:**

The results of the study showed that the local environment, which includes living conditions (no electricity, lack of availability of schools), social factors (isolation, national and local insecurity), working conditions (workload), the lack of financial compensation, and individual factors (marital status, gender), influences the attraction and retention of health professionals to work in rural areas. Human resources policies do not adequately take into account the factors influencing the retention of rural health professionals.

**Conclusion:**

Intersectoral policies are needed to improve living conditions and public services in rural areas. The government should also take into account the feminization of the medical profession and the social and cultural norms related to marital status and population mobility when formulating human resources management policies.

## Background

In 2006, the World Health Organization (WHO) characterized countries that did not have enough health professionals and were incapable of reaching 80% coverage of facility-based deliveries as having a “critical shortage” of personnel. Of the 57 countries in this situation, 36 were in sub-Saharan Africa [1].

Besides shortages, health professionals are unequally distributed between urban and rural areas. Rural regions are most affected, not only because of the shortage of health professionals but also because of the difficulties in retaining them in these zones [2, 3]. Many studies have focused on factors influencing the attraction and the retention of health professionals in rural areas in low- and middle-income countries [2, 4, 5]. A recent study on physicians and nurses (*n* = 15) in rural Bangladesh reported that difficult living conditions (absence of electricity and potable water), poor infrastructure, and lack of career development opportunities were the main factors impeding their retention [6]. In Vietnam, low salaries and problematic working conditions (limited equipment and transportation difficulties) were identified as the main factors discouraging health professionals from practising in rural areas [7]. Similarly, in Tanzania, a self-administered survey of 70 health professionals in rural areas reported that only eight were satisfied with their salary, two with equipment availability, and two with infrastructure. The study also identified lack of social support in the work environment as a contributing factor in demotivating health professionals in rural areas [8].

A few studies have explored why some health professionals are willing to work in rural areas [9–11]. However, those are essentially descriptive and quantitative and shed little light on factors influencing retention in rural areas [9–13].

Furthermore, studies on francophone African countries, most of which face critical shortages of healthcare providers, are scarce. Our research in Niger therefore aims to fill the knowledge gap on factors influencing the attraction and retention of healthcare providers in rural areas with a view to informing national programs and policies on health human resources. The findings could be extrapolated to other African francophone countries that share the same health system and social, cultural, political, and economic context as Niger [14].

One of the United Nations Sustainable Development Goals (SDGs) is to significantly increase the recruitment, number, and retention of health professionals in rural zones in francophone West African countries [15]. Our objectives were to understand the factors influencing the retention of health professionals in rural areas in Niger and particularly to identify and quantify the conditions in which midwifery students would be willing to practise in rural areas.

### Context

This research was commissioned by the Ministry of Health (MOH) of Niger, in West Africa. Niger’s population is estimated at 19.11 million, of whom 82% reside in rural areas [16]. It is one of the world’s poorest countries; 48.9% of its inhabitants live below the poverty line [16].

The organization of Niger’s healthcare system is vertical. At the top level are the national, regional, and university hospitals. The intermediate level is composed of regional hospitals, and the bottom (peripheral) level consists of district hospitals, integrated health centres (IHCs), and community health centres.

Life expectancy at birth is estimated at 61 years [16]. The estimated under-five mortality rate is 104 deaths per 100,000 live births [17]. The fertility rate is high, estimated at 7.6 children per woman [18]. The maternal mortality rate is high, with 630 deaths per 100,000 live births [19]. In 2014, the rate of facility-based deliveries was low (45.72%), as was the provision of emergency obstetric and neonatal care, at 20.3% [19].

Niger’s health human resources situation is characterized by a limited number of health professionals, especially nurses and midwives, and by unequal distribution (urban versus rural) in the territory. The ratios are one midwife per 4498 women of procreation age [20] and one nurse per 4159 inhabitants [21]. WHO’s Service Availability and Readiness Assessment project recommends a minimum of 23 core medical professionals per 10,000 population, which it defines as “physicians, non-physician clinicians, registered nurses, and midwives” [22]. In 2014, 75% of health professionals worked in urban environments, where only 16.2% of the population live. Only 13% of health professionals in obstetrics (nurses, midwives, senior technicians in gynaecology/obstetrics) worked in rural areas in 2015 [20]. For medical doctors assigned to rural IHCs, the State has established a standard of 3 years, which was not respected in more than 80% of cases, especially in the Dosso region [23]. This situation is similar in the whole national territory [23].

Several studies have been conducted locally to describe the professional culture of health professionals in Niger and their relationships with patients [24, 25]. However, factors influencing health professionals’ attraction and retention in rural areas have not yet been studied.

### Study site

Tillabery region, in the west of Niger, had an estimated population of 2,572,125 inhabitants in 2011 [20]. We selected the Tillabery region because it has a significant human resources problem compared to other regions, according to the MOH. Its main economic activities are agriculture, livestock farming, and fishing. The poverty indicator has risen slightly from 68.9% in 2005 to 71.7% in 2008.

The region is composed of six districts, with six district hospitals (DHs), 189 IHCs, and 428 basic healthcare centres. In terms of human resources, 71% of IHCs are managed by only one health professional and there is only one medical doctor per 100,000 inhabitants. Around 33% of the country’s total healthcare workforce is in its capital, Niamey, including about 40% of the midwives, while only 13% of the workforce practises in rural areas (19% of nurses and 8% of midwives) [20].

## Conceptual framework

**Fig. 1:**
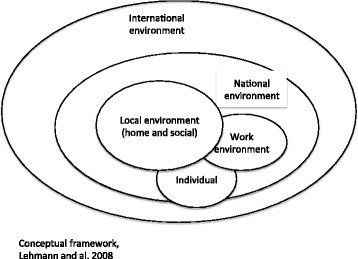
Conceptual framework

For this study, we used the framework developed by Lehmann and colleagues [2], which describes the factors that influence the attraction and retention of health professionals in rural areas. This framework was selected because it focuses on low- and middle-income countries (Fig. 1).

The framework has five dimensions: the international, national, local, and work environments and individual factors. Each consists of several factors that attract or discourage the health professional from practising in rural areas, which are interconnected.

The *international environment* includes factors of attraction, such as high salaries, good working conditions, and opportunities to develop one’s professional career in high-income countries.

The *national environment* includes factors of attraction and constraints, as well as general political climate (war, crime, conflicts, etc.), political and social stability, condition of public services, salary levels, and career opportunities in the country.

The *local environment* consists of general living conditions (housing, health services, schools, transportation, sanitation, electricity) and the social environment (degree of social isolation).

The *work environment* includes factors of attraction and constraints, as well as the management of health professionals, professional relationships, leadership practices, professional development opportunities, infrastructure, equipment, and job satisfaction.

*Individual factors* refer to the sociodemographic characteristics of the individuals, including their origin, age, gender, and marital status.

## Methods

### Research design

A mixed-method research design (exploratory and sequential) was used [26]. Preponderance was given to qualitative data because of the research question. The Mixed Methods Appraisal Tool (MMAT) was used to describe the whole research process [27].

The first stage identified the factors influencing the attraction and retention of the three categories of health professionals currently working in rural areas, through in-depth interviews and a documentary analysis. The second stage objectives were to identify and quantify the conditions under which midwifery students would be willing to accept positions in rural areas. This was done using concept mapping.

### Participants and sampling

Participants were recruited from three of the six districts of the Tillabery region (Tillabery, Ouallam, and Tera), selected because they were districts where the MOH considered the human resources problem to be most acute.

Three categories of participants were identified: (1) policy-makers and MOH officials, (2) health professionals (gynaecologists, midwives, nurses), and (3) local health actors (district management teams, locally elected officials, traditional leaders, non-governmental organizations (NGOs)).

Sampling was done by homogenization following the typology established by Pires [28]. This involves defining relatively homogenous groups but diversifying respondents within them. The aim is to recruit the greatest diversity of participants to obtain a wide range of perspectives. The internal diversification criteria were gender, years of experience in the profession, and professional practices in rural settings.

### Data collection

Data were collected using in-depth interviews, documentary analysis, and concept mapping.

The co-author (MM) and five research assistants conducted 163 in-depth interviews with policy-makers/MOH officials (*n* = 15), health professionals (*n* = 102), and local health managers (*n* = 46) (Table 1).

**Table 1:** Total number of participants of the study

Actors	Male	Female	Total
Policy-makers	10	5	15
Health professionals	59	43	102
Local health managers	43	3	46
Qualitative research total			163
Midwifery students		29	
Concept mapping total			29

An interview guide was developed and pre-tested, which covered the conceptual framework dimensions and sociodemographic data. Most interviews were recorded, with the participants’ consent; 29 (out of 163) were not recorded due to refusals—were mainly MOH senior managers. Systematic notes were taken during the interviews.

Documentary analysis focused on texts, policies, national strategies, legislation, and regulatory texts concerning human resources management in Niger (Appendix 1).

To understand the conditions under which midwifery students would be willing to practise in rural settings, we used concept mapping, a research method for obtaining group consensus on a given problem [29]. Midwifery students were chosen for this exercise because the problem of deployment and retention of healthcare professionals in rural areas particularly concerns midwives. The exercise involved 29 second- and third-year students from the Public Health and Social Action School in Niamey. The school of public health in the Tillabery region does not train midwives. The Niamey students are future midwives who will be solicited to work in rural zones.

In the first stage, participants were invited to complete the statement “I would be willing to work in a rural area for at least five years if….” A total of 94 completed statements were obtained. In the second stage, participants compiled them into categories relevant for them, then rated these statements’ importance on a 5-point Likert scale with 5 being most important.

### Data analysis

The interviews were fully transcribed and their content analysed [30]. Data were organized and analysed based on the conceptual framework dimensions. The documents were synthesized and analysed based on the research objectives. The concept mapping was analysed with statistical methods (hierarchical cluster analysis, then multidimensional scaling) carried out using an analysis module developed by PROVALIS (http://provalisresearch.com/).

The preliminary map and results were presented to the participants, who then named each cluster produced by the statistical analysis.

Diversifying the data collection methods and data sources enhanced the study’s internal validity through triangulation [31].

The qualitative and quantitative data are presented here in an integrated manner, as recommended by the MMAT tool [27].

### Ethical principles

The National Ethics Advisory Committee approved the research (Number 006\2015\CCNE). Authorization to conduct the study was obtained from the Tillabery region health authorities. Consent to publish the results was obtained from WHO and the MOH. Consent was obtained from all participants. The anonymity of the participants interviewed was maintained throughout the research process.

## Results

### International environment

In this study, no “brain drain” phenomenon was observed. No participant mentioned any desire to leave the country to work in a high-income country. Given the current international environment, we were surprised that this phenomenon, widely mentioned in the literature, was not raised. It would be interesting, in future research, to explore whether this situation is specific to Niger.

### National environment

Insecurity, low salaries, and poor financial compensation were identified as factors inhibiting retention in rural areas. With regard to insecurity, the current wars in Mali and Libya and the political instability in neighbouring Nigeria have made health professionals reluctant to practice in rural areas, especially in the north and near the Malian and Nigerian borders.

The problem of insecurity has overwhelmed us. Health workers do not feel safe working in these zones. (Immunization department, DH)

Salaries were a major concern for all participants but with different influence according to professional status. For civil servants, the main concerns were the lack of financial compensation for geographic distance and difficult working conditions in rural areas. Indeed, the medical doctor in charge of the rural IHC complained that physicians have effectively seen their salaries decrease by 50,000 FCFA (US$ 85) because they have been provided official lodgings similar to those previously assigned to the head nurses they replaced—a situation which they see as an injustice.

We’ve experienced unsuitable accommodations without water and electricity. And they’ve taken 50,000 francs for the accommodation, which isn’t even liveable. Even in urban centres, an accommodation worth 50,000 would be another thing. For someone they want to motivate to stay, it’s not normal. So, for the medical officers who are in the lodgings for managers, nothing can be taken for granted. Plus, the IHC isn’t fenced off (Head doctor, IHC)

Look at the Tillabery region. Even though it’s close to Niamey and it’s along the river, it’s a desert region. We don’t receive desert-area compensation like those in Agades, Tahoua, or Diffa. This situation is unfair (District head nurse)

In the concept mapping results, salary conditions (e.g., “larger bonuses in urban zones”) were classified as priority #2 out of 10. The statements “if we had a good salary” and “if the rural midwife’s salary were higher than that of an urban midwife” were rated 4.42/5 and 4.38/5, respectively (Table 2).

**Table 2:** The main conditions needed for midwifery students to practice in rural areas

**Statements**	**Priority**
**Respect midwives’ rights and duties**	**4.32**
**Better bonuses in urban zones**	**4.08**
**Zone favourable to family life**	**4.04**
**Total support for and safety of the midwives**	**4.04**
Security measures	3.73
Availability of qualified personnel and teamwork	3.65
Participation and collaboration of the community	3.64
Availability of necessary materials and equipments	3.54
Favourable living conditions	3.31
Accessibility to the zone and means of transport	3.24

### Local environment

The difficulties of accessing decent housing, electricity, drinking water, and schools for children were considered major obstacles.

Living conditions are tough. Roads are inaccessible. There’s no electricity or drinking water, and the markets aren’t equipped for buying the necessities (IHC Kandadji nurse)

Among professionals, accessing schools for their children was a significant problem:

Children’s education is a constant worry for health workers in rural areas. Sometimes, there aren’t any colleges, let alone a high school. For example, if I had a child who had to go to a college or a high school, I wouldn’t let them go to just any school. I’d need to look for a good school in Niamey. Each parent has this worry about his or her child’s future. There’s also the problem of having good tutors to leave your child with, and this has made some health workers refuse to stay and work in Tillabery. Here in Tillabery, which is the main town in the region, the schools aren’t of a high standard. Would someone who has a child in a private school in Niamey want to bring that child here? (Physician manager)

The midwifery students classified family circumstances as priority #3 out of 10. This statement group obtained an average score of 4.04/5. In this category, participants included their husband’s acceptance (4.17/5) and their children’s access to school in this locality (4.25/5).

However, the concept mapping results placed less importance on living conditions than did the qualitative data results, given that participants classified this priority as #9 out of 10.

Local insecurity was also a factor discouraging rural practice.

I’m robbed every day. It’s as if it’s an organized scam. They tell you someone is sick and you go to take care of them, and on your way back you notice you’ve been robbed. (Physician, Tillabery)

These difficulties were particularly worrisome for women:

Health workers are often women. They’re scared of living alone or being woken up at night. (Local mayor)

The midwifery students ranked security measures as priority #5 out of 10, in which they included their personal security, the geographic zone, and the workplace.

### Work environment

Poor infrastructure, inadequate equipment, and stock-outs of drugs and supplies were reported by health professionals as factors influencing retention in rural areas.

There’s a lack of materials to take care of sick people. Sometimes we have to ask for the leftover materials of the deceased. (Nurse, Tera DH)

At Ouallam, there’s a serious problem. The health facilities aren’t good. There’s a shortage of drugs. (Nurse, Ouallam DH)

Here at the IHC, we don’t even have an ambulance. If you had to do an evacuation, you wouldn’t even know what to do. (Head of an IHC)

Participants also perceived an overload of work due to insufficient health workers.

I’m alone in my IHC. Every day I do curative consultations, preventive consultations (prenatal and post-natal), and deliveries. People are always asking for me. There’s not even a health centre near us. (IHC supervisor)

The midwifery students divided working conditions into two categories: (1) the availability of qualified personnel and teamwork, classified as priority #6 out of 10, and (2) the availability of drugs, equipment, and infrastructure, classified as priority #8 out of 10.

Lastly, being assigned responsibility was the only attractive factor identified in all the results. This factor provided access to advantages such as housing, a motorcycle, and paid training and seminar opportunities (per diems).

When we name a worker as head of an IHC, they accept immediately without thinking, even if the IHC is far away or isolated. They know they’ll get more advantages. There’s the training with per diems. There’s official lodgings. In certain IHCs, you have electricity, drinking water, and you don’t pay to access it. You get a motorcycle…. So, the head IHCs in the bush are real heads. (EHC doctor)

### Individual factors

Gender and marital status were identified as individual factors negatively influencing the decision to work in rural areas. Local cultural norms dictate that women should follow their husbands, and not vice versa.

Women are protected by the “law” (informal). In this country, there’s a custom that says a woman should follow her husband. Because of this, today, we can’t send a girl to the bush, let alone a married woman. (Manager of a district)

The feminization of the nursing profession has also led to difficulties in recruiting, deploying, and maintaining female health workers in rural zones.

The predominance of women is the main problem. They don’t want to go to the bush because, once they’re married, we can’t send someone’s wife to the bush. (Manager)

(…) It’s feminization. Around 65% of our staff are female. We can have skilled women, but we can’t use them to our advantage because we can’t send them everywhere, such as in remote places where they would be very useful. What happens then is that we prefer that women practice in urban areas or in places with easy access (Manager of a district).

### Other factors

The concept mapping exercise identified other factors besides those in the conceptual framework. The 29 participants assigned greater importance to their professional and personal rights and duties. In fact, this category was classified as priority #1 out of 10 and received a score of 4.32/5. It encompasses working conditions (access to annual leave, maternity leave, leaves of absence, immediate hiring in public service) as well as professional development (access to ongoing training and respect for religious practices and professional autonomy). They also classified collaborating with the communities they serve as priority #7 out of 10.

### Inadequacy of policies related to retention of health professionals in rural areas

The policies implemented by the State are not aligned with the factors that influence health professionals’ attraction and retention in rural areas. In fact, the documentary analysis showed that health plans and strategy documents have been produced since 2003 to respond to the human resources crisis. These have focused on providing basic training and specialization in priority fields, encouraging on-site specialization, and recruiting qualified staff in partnership with the private sector and non-governmental agencies. For example, the 2005–2011 health development plan was aimed at developing skills, promoting the recruitment of contract workers, and offering incentive bonuses. However, the proposed actions did not target living conditions (housing, electricity, drinking water), social conditions, working conditions (management of health workers, equipment, financial compensation), and gender-based cultural norms affecting women’s mobility in rural zones. The documentary analysis also showed that the government has been aware of the unequal distribution of healthcare providers and the difficulties in retaining them in rural zones for a long time.

The results of this study showed that the attraction and retention of health professionals in rural areas are influenced by the local environment, which encompasses living conditions (electricity, drinking water, schools for children), social factors (isolation, international, national, and local security issues), working conditions (workload, numbers of health workers, availability of equipment, drugs, and supplies), salaries, financial compensation, and assigned responsibilities, along with individual factors such as marital status and gender (Table 3). The policies currently being implemented have little to do with the factors known to influence health professionals’ retention in rural areas.

**Table 3:** Summary of results regarding factors attracting and limiting retention of health professionals in rural areas

Dimensions	Explanation	Factors of attraction	Limiting factors
International environment	NA		
National environment			
Insecurity	Political instability in neighbouring countries (Mali, Libya, Nigeria)		++
Salaries and financial compensation	Low salary levels and absence of financial compensation incentives for geographic distance and living conditions in rural areas		++
Civil servant status	Obtaining tenure in a civil servant position from the entry level	++	
Career opportunities	Absence of career development opportunities		+
Local environment			
Living conditions	Poor infrastructures in roads, electricity, drinking water, means of communication, housing, and schools for children		+++
Social dimension	Social isolation, local insecurity, and disrupted family life (separation of couples, children)		++
Work environment			
Infrastructures	Poor equipment, technical services (ambulances, maternity leave), medicine, consumables		+++
Workforce and work overload	Limited number of health workers, excess quantity of work		++
Bonuses, promotions, responsibilities	Promotions, responsibilities, access to equipment (a motorcycle), ongoing training	+	
Respect for workers’ rights	Rights to annual leave, maternity leave, permission for absence		++
Individual factors			
Gender and marital status	Reluctance of married women to practise in rural areas: assignment of married women depends on their spouses due to family life, motherhood		+++
Respect for religious life	Full freedom to practice one’s religion		++

+++ Very important; ++ Important; + Less important

## Discussion

### Better living conditions: a fundamental condition for retention

Our results showed that living and family conditions were important factors in attracting and retaining health professionals in rural areas. These results concur with those of studies in numerous countries such as Ghana, Bangladesh, Ecuador, and Tanzania [2, 6, 32, 33]. Those studies have shown that difficulties related to housing, drinking water, electricity, and schools are the main obstacles to attracting and retaining health professionals in rural areas.

While the health professionals in our study indicated that poor living conditions were the determining factors in influencing them to not remain in rural areas, the midwifery students assigned greater importance to their rights as midwives (annual leave, maternity leave, leaves of absence, professional autonomy) as conditions for accepting rural postings. These results differed from those in a study in Ghana involving 238 third-year midwifery students, which used a quantitative research design suggesting two different scenarios [9]. Those students identified three factors that would motivate them to accept a rural posting: (1) a maximum 2-year assignment, (2) an adequate work environment (electricity, availability of technologies, drugs, and supplies), and (3) suitable housing. A qualitative study also conducted in Ghana using a focus group methodology showed that living and family conditions, professional life (working conditions, capacity building), and career advancement were the three main elements that would motivate students to accept a rural posting [34]. A quantitative study in Uganda with students in medicine (*n* = 246), nursing (*n* = 132), pharmacy (*n* = 50), and laboratory science (*n* = 57) showed that salary, working conditions, and management of professionals considerably influenced the choice to practise in rural areas [13]. The literature presents a wide range of conditions influencing health professionals’ willingness to work in rural areas. Therefore, further studies are needed to identify the most influential conditions. Health policies aimed at attracting and retaining health professionals in rural areas need to be adapted to the country.

### Feminization of the medical profession and gender norms

Two key findings had to do with the feminization of the nursing profession and women’s mobility and their consequences for the deployment, recruitment, and retention of professionals in rural areas. These two phenomena are intrinsically intertwined. As more females become healthcare providers, marital status and women’s mobility will play a greater role in the retention of healthcare providers in rural areas. These two phenomena were not the focus of our study but emerged as important factors.

The literature shows growth in the number of women in medical professions, especially in high-income countries such as the United Kingdom, Canada, and Japan [35–38]. In low-income countries, feminization of the medical profession has already started. To our knowledge, only one study has been published on this topic; that was conducted in three Portuguese-speaking African cities (Maputo, Praia, and Bissau) and concerned only medical doctors. The authors estimated that, of the 331 doctors working in those three urban zones, 42.6% were women [39].

In our study, feminization applies to the nursing profession in rural areas. In the Tillabery region, 42% of health professionals are female and 58% are male, while at the national level, 52.7% of health workers are male and 48.3% are female. In our study context, feminization of the nursing profession had begun and already affected health workers’ retention in rural areas. However, the extent of this feminization and its real impact on retention in rural areas are poorly explored and currently not well understood. More studies are needed to unpack this phenomenon [39].

The field of research on the influence of gender norms on health systems, and more specifically on the health workforce, is emerging tentatively [40]. According to Morgan et al. [41], “gender is defined as ‘the socially constructed roles, behaviours, activities and attributes that a given society considers appropriate for men and women’ [42]. Gender affects how females, males and people of other genders live, work and relate to each other at all levels, including in relation to the health system” [41].

Very little research has been undertaken to understand how gender norms influence male and female healthcare providers [43, 44]. Morgan et al. [41] suggest the following research questions: “To what extent is retention in rural areas more or less of a problem for female or male health workers? Is retention over time more or less of a problem for female or male health workers? Does this differ by marital status, parity and type of partner?”

### “Implementation gap” and policy implications

As mentioned, documentary analysis showed that several strategies were attempted but their implementation did not appear effective. Policies and plans, such as the Human Resources Development Plan (2011–2020), have been formulated without ever being put into practice, a phenomenon commonly observed in Africa and elsewhere [45, 46].

Besides not being effectively implemented, the proposed strategies do not sufficiently target living conditions, social factors, and individual factors. The case in Niger shows a discrepancy between the factors influencing health professionals’ retention in rural areas and the content of policies. Niger is not an isolated case. In fact, a documentary analysis revealed that similar policies in most countries do not adequately take into account the factors influencing the attraction and retention of health professionals in rural areas [2].

In Niger, policies need to focus above all on improving health professionals’ living conditions in rural areas by developing intersectoral policies to improve infrastructure, such as access to water, electricity, housing, security, and public services like schools. Furthermore, to retain health professionals in rural areas, strategies need to take into account the feminization of the nursing profession, as well as gender norms related to women’s mobility and marital status. Current policies seem to be gender-blind, meaning that the set of social roles, entitlements, responsibilities and obligations associated with being a female is currently ignored [47].

### Study strengths and limitations

This study’s strength resides in its mixed-method design (qualitative and quantitative) and in the diversity of data sources (diversification of actors) and data collection methods (concept mapping, interviews, documentary analysis). The data were collected by a research team familiar with qualitative research methods and with both the context and the local language.

However, this study has limitations. Social desirability bias could have been introduced during the concept mapping exercise with the midwifery students. To reduce this bias, participants were assured of the anonymity of their responses. They were told that the aim was to understand the conditions under which future midwives would be willing to work in rural areas and that their views were important for influencing policies on human resources management. Another limitation of this study is that the concept mapping was carried out with students not yet working in rural areas. As such, their statements were based on expectations and not on actual experience in rural areas. Finally, the collected data were limited to three districts and one training school. Consequently, any extrapolation of the results should be done with caution.

## Conclusion

This study showed that many complex and interrelated factors at different levels (national, local, individual) influence the attraction and retention of health professionals in rural areas in Niger. The most influential factors explaining health professionals’ reluctance to practise in rural areas were adverse living conditions and individual factors related to gender and marital status. The results of the qualitative research and the concept mapping were almost the same.

With regard to policy implementation, the policies did not sufficiently take into account the factors influencing health professionals’ decision to work in rural areas. Intersectoral policies are needed that will mobilize several government departments to improve living conditions, social factors, and working conditions of health professionals in rural areas. When formulating human resources policies, Niger’s government also needs to consider the feminization of the nursing profession and the social and cultural norms related to marital status and women’s mobility.

## Appendix 1

**Table 4:** Documents consulted

Documents			Importance
Policy documents			
National health policy		MSP/LCE, May 2002	Presents the main outlook and the approach of national authorities in the health sector
Health development plan 2005–2010		MSP, Cabinet meeting, 18 February 2005	
Health development plan 2011–2015		MSP, Cabinet meeting, 21 January 2011	
Human resources development plan 2011–2029		MSP, September 2011–2020	Analyzes the human resources situation in health and develops the different strategies and interventions to implement for the period 2011–2029
Health indicators. Presentation PP of DEP at the National Council of Health meeting		MSP	Presents the monitoring indicators of PDS, region by region
Strategic management plan for human resources in the context of decentralization		MSP/SG/DGR/DRH	
Report studies, audits, evaluations			
Final organizational and functional audit report of MSP		MSP, OMS, CTB/PAI 2015	
Health and poverty in Niger. Towards the MDGs		World Bank, March 2004	Contextual setting: the region of Maradi
Study on the motivation to practice in rural areas		MSP; SEDES-Sarl, June 2005	
Audit report from DRSP		MSP	
Survey on the satisfaction of the needs of beneficiaries of healthcare services		MSP, Cabinet Bozari, September 2009	
Legislative and regulatory texts			
Text	Introduction date		Compensation and targets
Decree 79-147	20-09-1979		Isolation pay Health personnel
Decree 86-124	11.01.1986		Rolling compensation Surgeon general of a department or wing
Decree 92-032	17.01.1992		Telephone allowance Hospital directors and CHD, surgeons, gynecologists, financial officers of national hospitals and maternity wards, hospital administrators, health department directors, medical supervisors, department or wing doctors, family health officers of CHD and CM
Decree 94-196/PRN/MSP	10.12.1994		Housing allowance Doctors, pharmacists, dental surgeons (if the officer is not housed)
Decree 97-141	20.03.1997		Work bonus Civil servants occupying a position or performing health care in hospital and non-hospital health facilities
Decree 98-036	23-01-98		Premium suggestion Risk allowance Personnel occupying positions in hospital and non-hospital health facilities
Decree 2006-079/PRN/MSP/LE			
Decree 2012-61/PRN/MSP/MF/PF/PE/MFP/T/MF	29 February 2012		
Decree n°2012-500/PRN/MFP/T/MF	05 November 2012		Transportation allowance and housing allowance Public service premium Government officials and civil servants^a^
Decree n°2013-053/PRN/MFP/T/MF	13.02.2013		Childcare fees and damages, (civil servant managers occupying hospital and non-hospital positions) bonus incentives to officers (health executives and social workers practising outside of Niamey)

^a^In terms of this decree, government officials means, civil servants, public sector contract workers and auxiliary agents of the state. (Article1)

## Abbreviations

DH: District hospital
EHC: Epidemiological health centre
FCFA: Francs CFA
IHC: Integrated health centre
MMAT: Mixed Methods Appraisal Tool
MOH: Ministry of Health
NGO: Non-governemental organization
SDGs: United Nations Sustainable Development Goals
WHO: World Health Organization
